# Peer-assisted teaching student tutors as examiners in an orthopedic surgery OSCE station – pros and cons

**DOI:** 10.3205/iprs000096

**Published:** 2016-07-14

**Authors:** Peter Melcher, Dirk Zajonz, Andreas Roth, Christoph-E. Heyde, Mohamed Ghanem

**Affiliations:** 1 Department of Orthopaedic Surgery, Traumatology and Plastic Surgery, University Hospital Leipzig, Germany

**Keywords:** student tutor examiners, peer-assisted teaching, orthopaedic surgery OSCE

## Abstract

**Background:** The OSCE (objective structured clinical examination) is composed of oral and practical examination in order to examine students’ abilities to imply clinical examination techniques and to interact with patients. The examiners for this procedure can be either lecturers or peers. The aim of this work is to evaluate the peer-assisted teaching student tutors as examiners in an orthopedic surgery OSCE station.

**Methods:** We analyzed the OSCE data from 2013 to 2015. During this period over 300 medical students were examined each year. An evaluation was conducted at an orthopedic station and examined by peer students to assess the advantages and disadvantages of peer-assisted teaching student tutors as examiners.

**Results:** We have noticed that student peers are more flexible regarding their schedule and they have been well trained for OSCE. Concerning the economic aspects, student peers are clearly of major economic advantage.

Disadvantages were not reported in our study probably because peers were well trained and the checklists are monitored regularly.

**Conclusion:** Student peers in OSCE are of major advantage due to their flexible time schedule and relatively low costs. They must be well trained and the checklists are to be monitored regularly. Our study shows that peer tutor examiners conducted the examination as competent as lecture examiners. However, legal restrictions on the employment of students should be considered.

## Zusammenfassung

**Hintergrund:** Ein OSCE (objective structured clinical examination) ist zusammengestellt aus mündlichen und praktischen Prüfungen um die studentischen Fähigkeiten der klinischen Untersuchung und des Umgangs mit den Patienten zu beurteilen. Als Prüfer können dabei Peers und Dozenten eingesetzt werden. In dieser Arbeit werden studentische „Skills-Lab“-Tutoren als Prüfer einer OSCE-Station Orthopädie evaluiert.

**Methoden:** Wir analysierten die Zahlen der OSCEs von 2013 bis 2015. In jedem Jahr wurden mehr als 300 Studenten geprüft. Die Prüfungsergebnisse nach Einsatz von studentischen „Skills-Lab“-Tutoren als Prüfer einer OSCE-Station Orthopädie werden ausgewertet.

**Ergebnisse:** Die Auswertung der studentischen Peers in unserer Studie zeigt, dass sie flexibler in der zeitlichen Planung sind und gut auf den OSCE vorbereitet waren. Zudem haben studentische Peers einen klaren ökonomischen Vorteil.

Nachteile wurden in unserer Untersuchung nicht festgestellt, was mit dem Fakt zusammenhängen kann, dass die Peers gut vorbreitet wurden und die Checklisten regelmäßig überprüft werden.

**Schlussfolgerung:** Studentische Peers im OSCE haben große Vorteile durch ihre zeitliche Flexibilität und relativ geringe Kosten. Sie müssen gut vorbereitet werden und die Checklisten müssen regelmäßig geprüft werden. In den ausgewerteten Prüfungsergebnissen zeigt sich kein Unterschied zwischen studentischen und ärztlichen Prüfern. Die gesetzlichen Grundlagen für den Einsatz von Studenten sollten bedacht werden.

## Introduction

According to the German Federal Medical Training Regulations revised in 2002 [1], on-the-job training should play a more important role in medical education. This requirement is comprehensible because of the huge growth of theoretical knowledge. For that reason, the imparted practical skills may have lost their importance in medical education. There are a number of different approaches to meet this requirement and to combine theoretical and practical education. The universities for example introduced problem based learning (PBL) [2], [3], [4], courses in skills labs [5] or OSCEs [6] to their education program. The examination of the acquired skills has to be objective and comparable, which is difficult to ensure with normal examination procedures used in Germany, for example the multiple choice test. One possibility to examine skills such as examination techniques and to prepare the students for the practical state examination is the OSCE.

An “Objective structured clinical examination” (OSCE) comprises an oral and a practical examination. Harden et al. first introduced the OSCE in 1975 [7]. To pass the OSCE the examinees have to complete one course out of many different stations with a time limit of 5–10 minutes [8]. In this kind of examination simulators and simulation patients are used in order to valuate examination techniques and communication with the patient. Checklists are used for the evaluation. Hofer et al. showed that the OSCE is strongly accepted among young doctors [9] and medical students [10] with growing popularity in Germany. 

We evaluated the advantages and disadvantages of peer tutor examiners in a team out of skills lab employees and orthopedic lecturers and tried to conclude if peers could act as examiners in an OSCE.

## Methods

At the medical faculty of Leipzig different OSCEs are performed. In the 5^th^ semester, all students have to pass through an examination course in order to learn basic examination techniques of many different medical departments like internal medicine, otorhinolaryngology, dermatology, orthopedics and many more. At the end of the course, they have to pass an OSCE.

We analyzed the results of the OSCE from 2013–2015. In each year, over 300 students were examined. All students had to pass 1 out of 3 courses with 5 stations. The course consisted of one station internal medicine and 4 randomized stations out of 8 departments. Each station has to be attempted within a time limit of 5 minutes. Some stations were examined by lecturers, some by peers. Lecturers are trained physicians at the university medical center Leipzig, who examined the station of their faculty. The peers are special trained tutors of the Skills-Lab Leipzig. They are didactically trained for communication with students examinees. They get a special training in preparation of the OSCE with the director of the Skills-Lab Leipzig and the lecturer of the responsible department. The tutors learn about the special procedure, tasks and behavior as a peer tutor examiner.

All peer examiners are employees of the Skills-Lab Leipzig. They are examining the same stations in OSCE that they are already acquainted with. So they are trained in the examination techniques and handling of the simulators. In addition, they get instructions how to act and behave in an OSCE and every peer examiner is mentored by the responsible lecturer.

The peer tutor examiners get the normal student assistant pay. 

We compare the results of the station examination of spine and pelvis, examined by peer tutors, with the station clinical examination of knee or shoulder, examined by lecturers.

Concerning peer tutor examiners and regarding the principle of objective examination, the checklists are of major importance. There are many different ways of checklist design and scoring. We monitored the checklists used for the OSCE in Leipzig and found that the points at the checklists have to be as clear as possible; otherwise, the reliability is questionable. An example how we structure our checklists is shown in Table 1 (Tab. 1).

The skills, which the students have to demonstrate, are the testing of the range of motion in the thoracic und lumbar spine including the documentation by using of neutral null method including the Schober’s test and Ott’s sign. They also have to perform the Trendelenburg’s sign and examine the pelvic position. Simulation patients were employed. These simulation patients have medical background and get training on how to act during the examination. Most of them are tutors of the Skills-Lab Leipzig and some are medical students. To pass the exam the students had to accomplish at least 60% of the maximal points on the checklist. 

## Results

Within the 3 years of observation, between 306 and 324 students participated each year in the OSCE course. One third had to complete the Station “Examination of spine and pelvis” which is equal to 98 to 105 students. 0% to 1.3% failed this station, which is a nearly similar result to the station for knee and shoulder examination shown in Table 2 (Tab. 2).

The total failure rate only includes the real number of students who failed. That means students, who did not pass more than one station only count once. 

## Discussion

**Figure 1:**
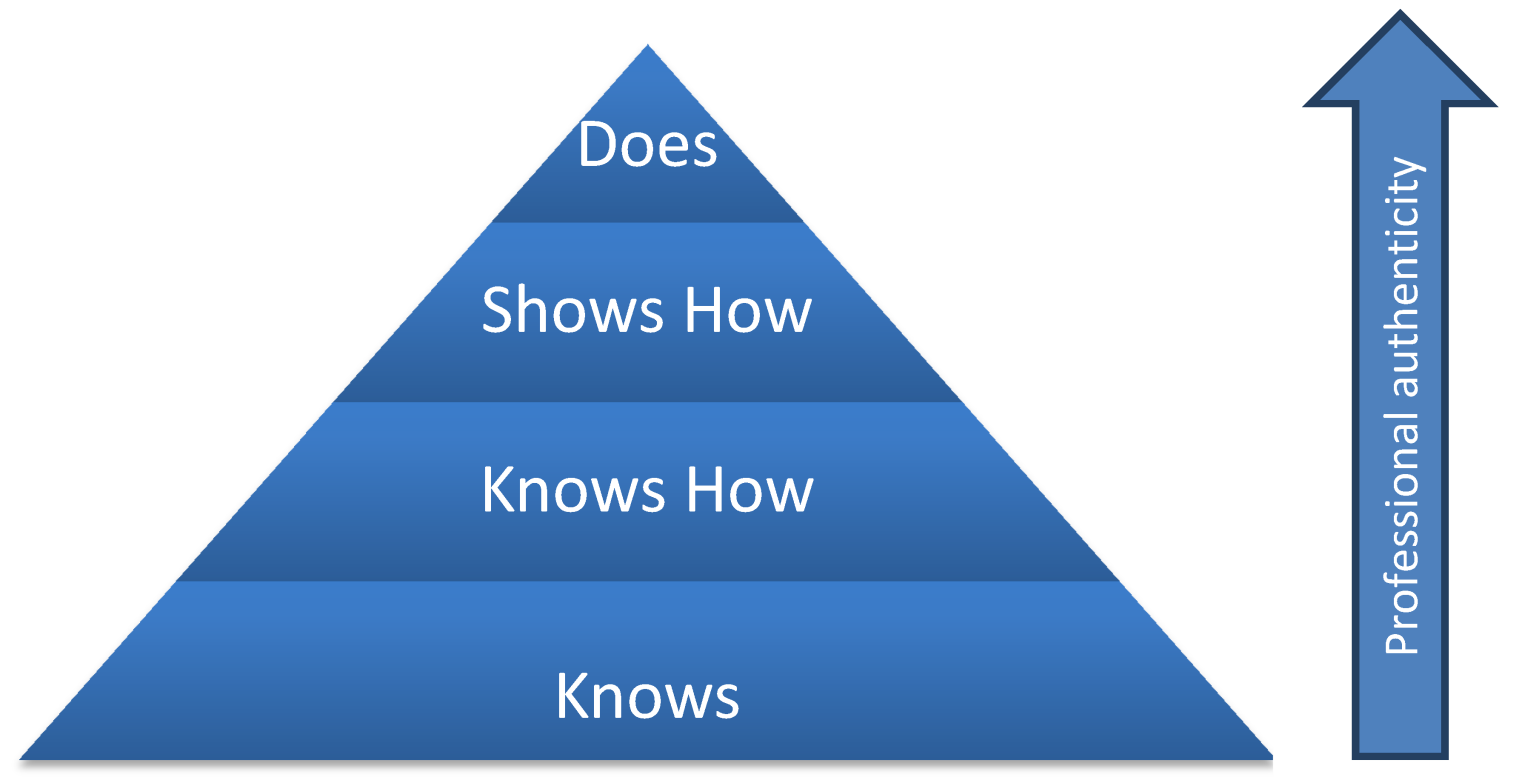
Miller pyramid [11]

There are different levels of clinical competences like Miller has shown in 1990 with the Miller pyramid [11] shown in Figure 1 (Fig. 1). Most examination procedures in medical education are testing the first two levels of these competences. To examine the next level and satisfy the actual requirements of the German Federal Medical Training Regulations there must be other examination procedures than the normal multiple-choice test, which is mostly used in Germany. The OSCE should play a bigger role because it has very good preconditions for testing the levels of the Miller pyramid [12].

After 3 years of conducting this OSCE in Leipzig we analyzed advantages and disadvantages from the peer tutor examiners and lecturers standpoint. As Carpenter and Chenot showed in a literature review analyzing the subcategorized costs, the financial effectiveness of OSCE [13] is given. For further cost reduction it makes sense to employ peer examiners because they have much lower cost compared to trained physicians and lecturers. Further, student peer examiners have more flexible time schedule and therefore a better opportunity to be trained as examiners specifically for OSCE. With this training, the peers are better prepared for OSCE and not influenced by other methods of examination. Most times the lectures have to work in hospital and afterwards they need to be trained for this examination procedure. There is no doubt that peers have to be trained for their task [14]. A good method to prepare the examiners is to use videos [15]. Burgess et al. showed that peer examiners also learn from their assignment which is a good side effect [14]. In this context, the legal aspects also have to be considered. In Germany, there are differences among the federal states about the fact whether peer examiners are authorized to examine. 

Whether peers can be employed as examiners, has to be verified by every single institution of education [16]. 

It has to be considered that there might be a difficult relationship between peers, especially if the examiner is “academically” younger than the examinee. The way to handle this has to be taught during the peer examiner training. Further, the inter-examiner comparability between peers and lecturers has to be considered. Yet, providing a good checklist, which has to be monitored every year, ensures an objective and good examination.

We realize that it is difficult to compare 2 different stations and come to a reliable conclusion but in our opinion the results of the OSCE shows that peer tutors could act successfully as examiners. The influence of peer examiners on OSCE is still up-to-date [17], [18], though we plan further prospective survey.

The results show a failing rate at the orthopedic station of 3% that might seem a bit low and could have many reasons. In our opinion, our standardized checklists and well-trained examiners help the students preparing and performing the exams and thus contribute to favorable results.

Our analysis has shown that there has been a relatively high total failure rate in 2014, which might exist because the students knew that failing the exam would result in attending a course in the Skills-Lab Leipzig (Lernklinik Leipzig). In 2015, the consequences of failing changed. Since 2015, a re- examination held by the lecturer has to be passed in every failed station, which could be an explanation why the failure rate dropped in 2015.

## Conclusion

An orthopedic OSCE station is a reliable method to evaluate basic examination skills. Lecturers and peer tutor examiners are examining with equally good performance. 

Student peers in OSCE are of major advantage due to their flexible time schedule and relatively low costs. They must be well trained and the checklists are to be monitored regularly. However, legal restrictions on the employment of students should be considered.

## Notes

### Competing interests

The authors declare that they have no competing interests.

## Figures and Tables

**Table 1:**
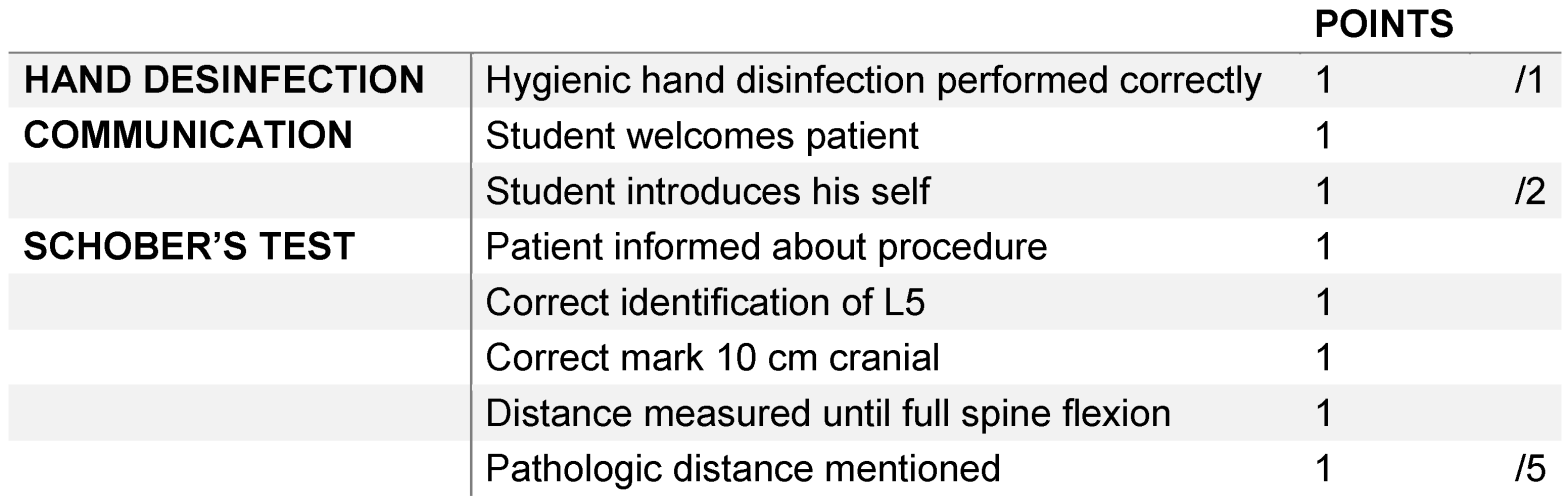
Part of a checklist showing how checklists are structured

**Table 2:**
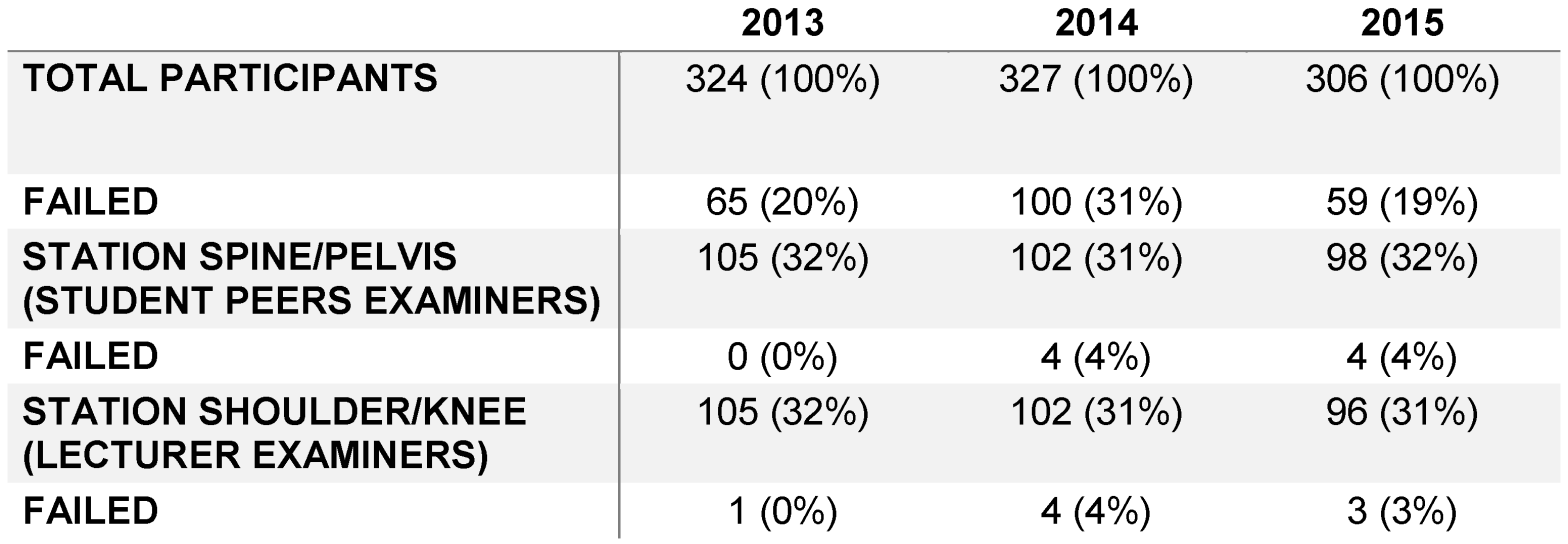
A comparison of the examination results of OSCE
